# An Improved Capsule Network for Image Classification Using Multi-Scale Feature Extraction

**DOI:** 10.3390/jimaging11100355

**Published:** 2025-10-10

**Authors:** Wenjie Huang, Ruiqing Kang, Lingyan Li, Wenhui Feng

**Affiliations:** Main Campus, Automation College, University of Science and Technology Beijing (USTB), Haidian District, Beijing 100083, China; m202320797@xs.ustb.edu.cn (W.H.); m202310527@xs.ustb.edu.cn (L.L.); m202420860@xs.ustb.edu.cn (W.F.)

**Keywords:** capsule network, multi-scale feature extraction, image classification, custom convolution, attention mechanisms, Dense Block

## Abstract

In the realm of image classification, the capsule network is a network topology that packs the extracted features into many capsules, performs sophisticated capsule screening using a dynamic routing mechanism, and finally recognizes that each capsule corresponds to a category feature. Compared with previous network topologies, the capsule network has more sophisticated operations, uses a large number of parameter matrices and vectors to express picture attributes, and has more powerful image classification capabilities. However, in the practical application field, the capsule network has always been constrained by the quantity of calculation produced by the complicated structure. In the face of basic datasets, it is prone to over-fitting and poor generalization and often cannot satisfy the high computational overhead when facing complex datasets. Based on the aforesaid problems, this research proposes a novel enhanced capsule network topology. The upgraded network boosts the feature extraction ability of the network by incorporating a multi-scale feature extraction module based on proprietary star structure convolution into the standard capsule network. At the same time, additional structural portions of the capsule network are changed, and a variety of optimization approaches such as dense connection, attention mechanism, and low-rank matrix operation are combined. Image classification studies are carried out on different datasets, and the novel structure suggested in this paper has good classification performance on CIFAR-10, CIFAR-100, and CUB datasets. At the same time, we also achieved 98.21% and 95.38% classification accuracy on two complicated datasets of skin cancer ISIC derived and Forged Face EXP.

## 1. Introduction

In the realm of deep learning image classification, complicated models can typically reflect more accurate picture feature information than other models [1]. With the deepening of layers in CNN and the growth in convolution computation, the basic faults of traditional CNN in structure are increasingly exposed. In CNN, features are represented by scalars. When features travel from the bottom layer to the high layer, the high layer cannot obtain the spatial relationship between various features through scalars [2]. As the number of layers deepens, the spatial relationship between different characteristics becomes increasingly complex, resulting in substantial feature loss at the bottom of the pooling layer. In certain practical contexts, CNN has shown deficiencies in spatial understanding [3]. The capsule network (CapsNet) suggested by Hinton et al. handles this problem successfully [4]. CapsNet is based on the workings of the human visual system, which consists of a hierarchy of “Capsules” that process visual information at increasing levels of abstraction to extract more abundant details of the picture target. It has been proven that compared with other CNNs, the capsule network offers advantages in numerous image classification application scenarios, with higher classification accuracy [1,5,6] and a more efficient network routing method [7,8].

The capsule network is built of capsules and dynamic routing. By removing the pooling layer in CNN and substituting the pooling function with a combination of vector representation and dynamic routing, the problem of spatial information loss can be handled [9,10]. Compared with the ordinary routing technique, dynamic routing utilizes a more flexible parameter coupling mechanism, resulting in a more complicated structure and higher total computing complexity than other CNNs. Taking an image experiment on the CIFAE-10 dataset as an example, the CapsNet calculation of the three-layer structure is larger than that of the 1202 layers [11]. Owing to the challenge of computational complexity, capsule network study demands high-performance hardware equipment, resulting in fewer relevant studies than other networks. CapsNet is generally lagging in the research in the field of deep learning; for example, several improved approaches have been widely utilized in other networks [12,13,14,15] but sometimes only exist at the theoretical level in capsule networks, missing detailed practical data support [16,17]. To accomplish the task of picture classification with higher accuracy, some researchers expect to improve its accuracy by directly fusing more models under suitable hardware conditions. In CIFAR-10, other model structures were fused based on the conventional CapsNet. When the fusion model is increased from 1 to 4, the accuracy is increased from 69.34% to 71.5%; nevertheless, the accuracy rate still maintains 71.5% when seven models are fused on this basis [18]. The preceding results suggest that the quality of CapsNet image classification cannot be enhanced merely by fusing more models.

To overcome these challenges, scholars have conducted improvement work in numerous directions. By incorporating different network architectures to generate a smaller number of primary capsules of greater quality, Hu et al. enhanced the dynamic routing speed by three times and reduced the amount of calculation of the subsequent capsule layer [19]. However, the network relies largely on the fusion model, which requires ongoing modification of the structure and parameters of the fusion model to adapt to diverse datasets. Chen et al. employed a dot-product-based attention method to generate a completely connected graph between capsule layers [20]. This strategy improves the accuracy of the model by highlighting the form of key capsules; however, it considerably increases the quantity of calculation, making the basic training period climb to two times that of the traditional capsules. Although some research has made significant progress, the present improved capsule network model typically has some problems: (1) It is difficult to reduce the quantity of calculation and enhance accuracy at the same time. (2) Optimizing performance on several datasets simultaneously is tricky.

In summary, the capsule network is a network with spatial understanding capacity, which has better understanding and classification ability for the location, shape, and other aspects of the target in the image. However, there are certain challenges in its application, such as the vast quantity of computations, difficulty improving model research and practice, and lack of scientific experimental data backing for some hypotheses. These difficulties have hindered the development of capsule networks in the past. Although some studies have been improved, the quantity of calculation and accuracy of an improved capsule network picture categorization still need further optimization. In addition, the present upgraded capsule network can only increase the accuracy of basic datasets; however, it is difficult to play its function in complicated datasets with vast images and many categories. The overall generalization of the revised CapsNet model was unsatisfactory.

## 2. Improved Network Design

**Figure 1:**
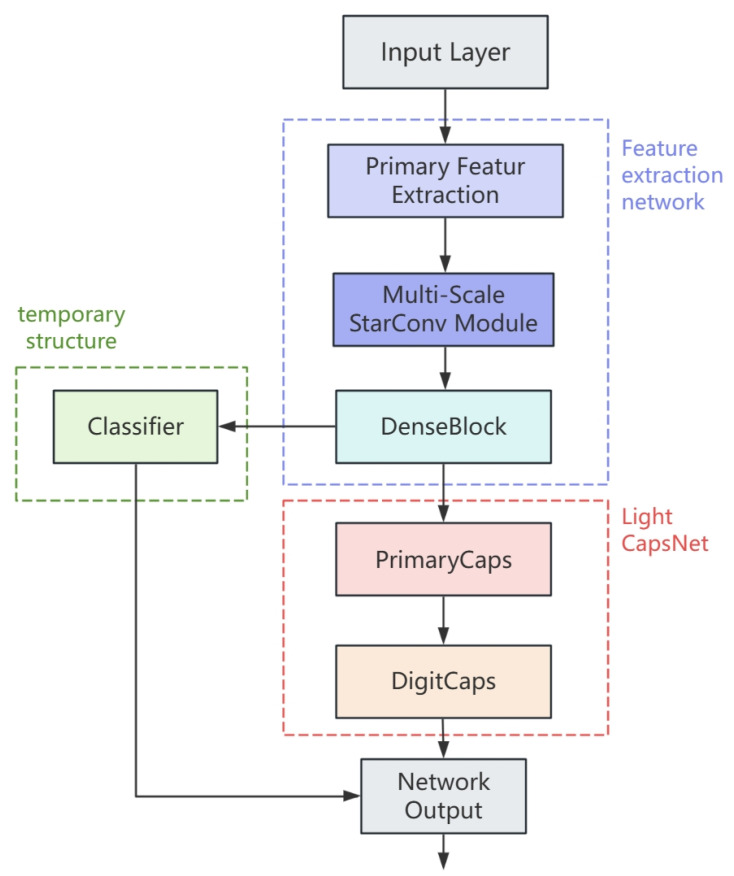
Improved multi-scale capsule network structure. The network consists of three pieces and has two information paths. One path is through the feature extraction network and the temporary structure; the other path is through the feature extraction network and the Light CapsNet.

Aiming at the difficulties of inadequate generalization, high computational complexity, and insufficient accuracy improvement in the current capsule network, this research suggests a multi-scale feature improved capsule network based on StarConv. The focus on improvement of the improved multi-scale capsule network is to introduce a special multi-scale feature extraction module based on star convolution (StarConv) to achieve the balance between network classification ability and computational overhead and improve the performance of the capsule network. The updated model structure is depicted in Figure 1. The full network is broken into three parts: feature extraction network, Light CapsNet, and temporary structure.

The feature extraction network is made of initial convolution (Conv), star convolution multi-scale feature module (StarConv-SEMS), and dense connection block (Dense Block). In this part, a set of ordinary convolutions are preferentially employed for feature extraction, and then the generated feature map is input into the multi-scale feature extraction module with StarConv as the main structure. In order to further improve the quality of features, some attention mechanisms are integrated into the multi-scale module. Finally, the feature transfer mode and the output channel are optimized by the dense connection block to ensure that the feature can be used as the input of the future capsule network structure. This component is the essential optimization of the upgraded capsule network. Through the initial convolution processing, the input image is fixedly extended into a three-channel 32 × 32 image and matches the input requirements of the low-pixel image of the capsule network. After the image enters the feature extraction network, the number of channels is expanded from 3 to 128. The precise features contained in the image are abstracted and the feature expression becomes rich, which can be used for subsequent calculation and screening. At the same time, in order to reduce the overall computational complexity of the model, bilinear interpolation is employed to achieve smooth down-sampling of feature maps. Then, the feature map size is compressed from 32 × 32 to 24 × 24. To compensate for the loss incurred by down-sampling, 1 × 1 convolution is added; the final output size of the feature extraction layer is 480 × 24 × 24.

The temporary structure is formed from an auxiliary classifier. The function of the temporary structure is to directly extend the output of the feature layer into the vector set of the relevant dimension according to the number of categories of the current image classification dataset. Combined with the related evaluation method and loss function computation, the output may immediately show the image classification results of the current network. This section and the feature extraction network compose the first complete path of the network, and the picture categorization is completed without the participation of the capsule network. The temporary structure is considerably simple, and the computational complexity is significantly lower than the capsule network, but it is difficult to satisfy high-precision image classification needs because of its weak feature expression ability.

The capsule network topology described in this study continues the classic composition of the primary capsule (PrimaryCaps), the digit capsule (DigitCaps), and the intermediate dynamic routing of the capsule network. In the network design, the network parameter is decided to be set to 4704 × 32, where 4704 represents the corresponding number of primary capsules and 32 denotes the dimension of each capsule. The number of digit capsules is equal to the number of species in the current image classification dataset. In the training of capsule network image classification, a digit capsule represents the features of a category. This method is more granular than other deep learning networks where one vector represents one class. On the basis of this architecture, a light CapsNet is introduced, and the matrix decomposition approach is employed to minimize the parameter composition of dynamic routing calculation. At the same time, the Top-K mask mechanism is incorporated in the dynamic routing, and the parameter is set to 0.7 to reduce the calculation loss of noncritical capsules. The above optimization can lower the training time and memory usage without compromising the output quality of the capsule.

### 2.1. StarConv Multi-Scale Feature Extraction

**Figure 2:**
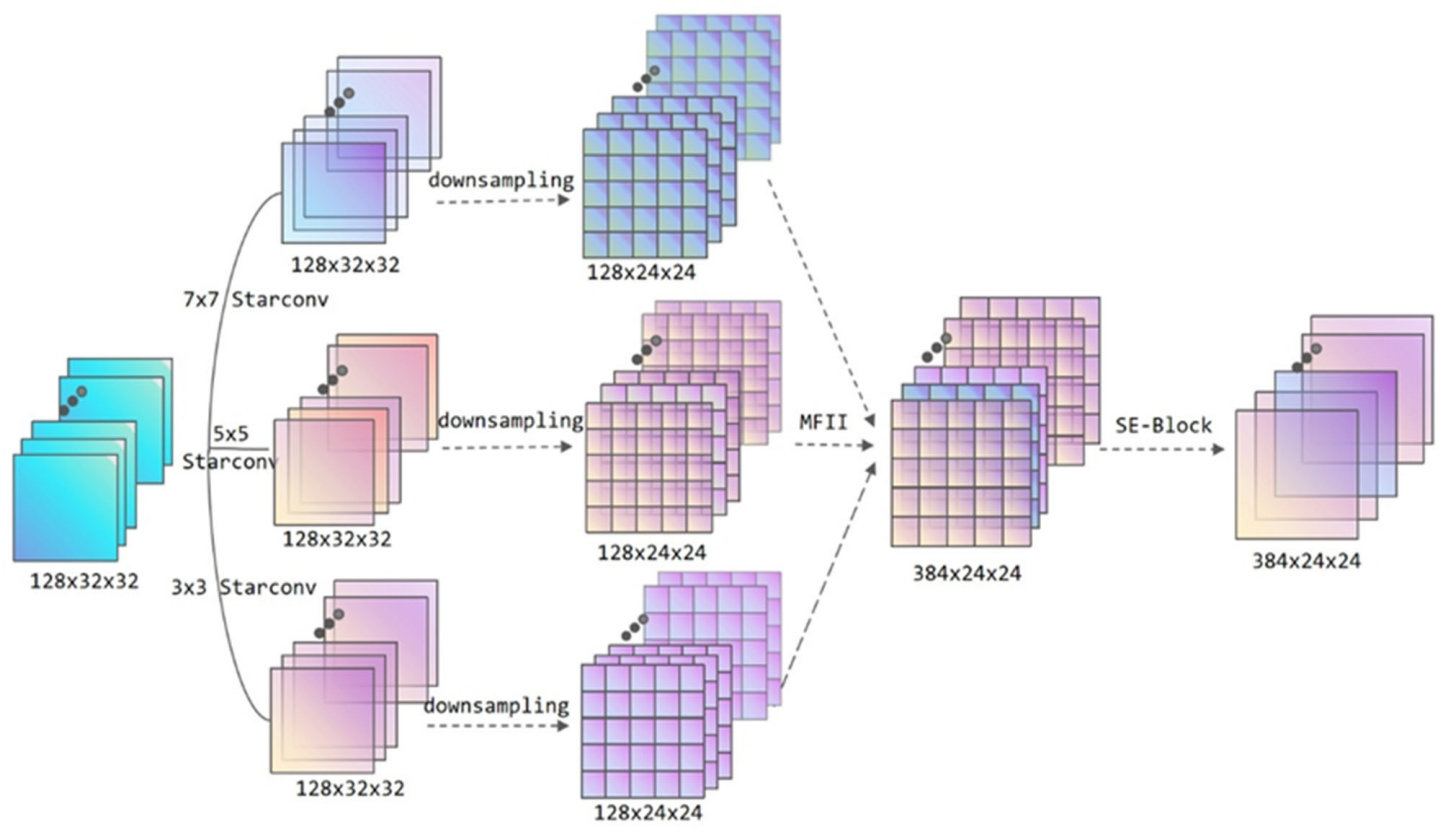
StarConv multi-scale feature extraction module structure. The module employs three distinct dimension sizes of StarConv: after extracting the features of various dimensions, using MFII method for feature fusion, and optimizing the output using the SE attention mechanism.

In this paper, an enhanced multi-scale feature extraction module based on star topology convolution is proposed. Multi-scale feature extraction is a form of convolution or other feature extraction structure that adds several different dimensions in the input phase to extract numerous features of different dimensions; then, these features are organically fused to provide a richer feature expression approach. In this study, the design of the multi-scale module combines three-star convolutions with sizes of 3 × 3, 5 × 5, and 7 × 7 to construct the multi-scale feature extraction module, and the complete structure is depicted in Figure 2. Through this module, the input feature is enlarged from the original 128 channels to 384 (128 × 3) channels, and the size of the feature map is compressed to 24 × 24. The feature expression becomes more plentiful, which is favorable to the further picture classification operation.

**Figure 3:**
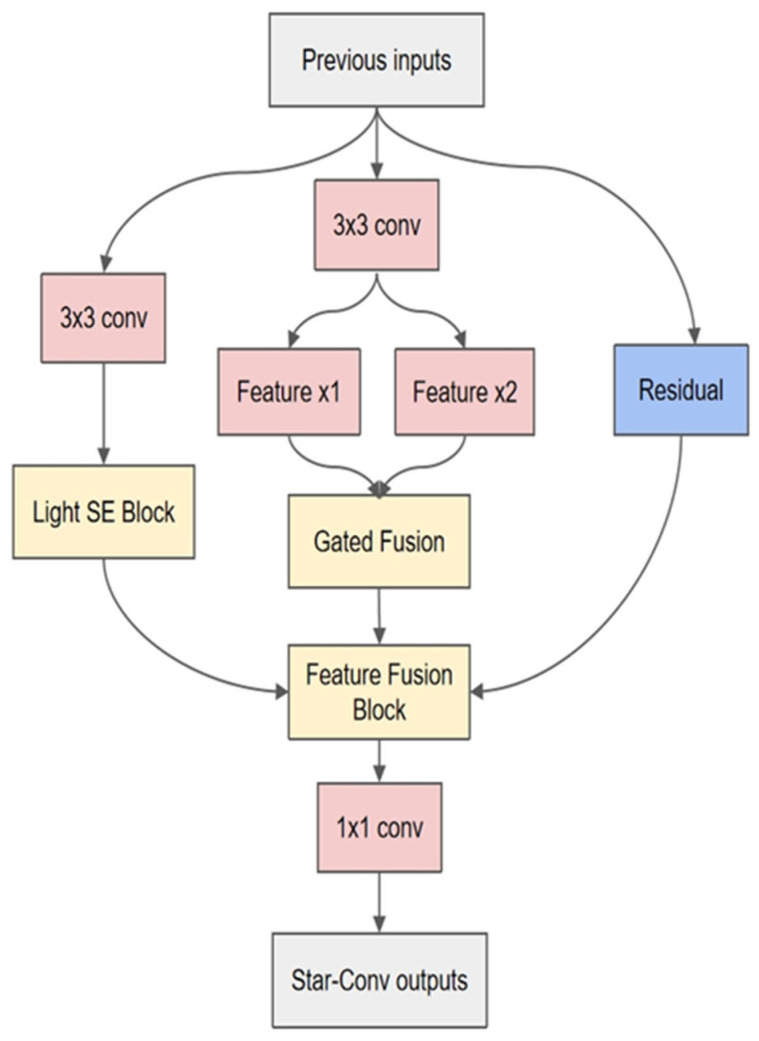
Schematic diagram of StarConv structure (taking the structure of 3 × 3 as an example).

Star topology is a typical kind of network organization, and its basic feature is that several peripheral nodes converge to the same central node by direct connection. In the field of deep learning, the topology is also introduced to optimize the structural complexity of particular models, so as to obtain more efficient computation and parameter management [15]. The convolution kernel connection mode of star topology convolution (StarConv) can be horizontal, vertical, and diagonal, which is a method to optimize the full connection into a star connection by decomposing the large convolution kernel into multiple small branches and to improve the efficiency of star connection, methods for reducing overfitting risk and computational complexity. Under this strategy, the classification ability of the original model is nearly unaltered, while the computational cost can be greatly decreased. The multi-scale feature extraction layer in this paper is based on a custom star-structured convolution, which uses multiple feature processing methods in a single convolution, decomposes the features into different channels for processing separately, and uses the multi-scale feature extraction layer to extract the features; after that, the features are refused, and the structure is adapted to the multi-scale design idea. The custom StarConv structure designed in this paper is depicted in Figure 3. The StarConv contains three channels, which are Light SE Block, Feature Gated Fusion, and Residual connection. The above three feature-processing channels have the characteristics of light weight and high efficiency. The input is processed in different ways, and finally the features are stitched and fused.

An attention mechanism is a deep learning strategy that focuses more on effective information and ignores unnecessary information in picture classification tasks. According to the characteristics of a capsule network with strong spatial comprehension ability, we can choose to reduce the computational overhead on the attention mechanism and prevent the model redundancy by adopting the lightweight attention mechanism. The Squeeze and Excitation (SE) attention technique enables the model to automatically learn the weights of distinct channels and suppress the feature expression of irrelevant channels. At the same time, it will not clash with the feature needs of the succeeding capsule network and can better play the role of the network, lowering the influence of added modules on the overall calculation. On the premise of the fundamental SE mechanism, part of the calculation is fine-tuned, and the final complete attention mechanism calculation procedures contain three steps: compression, incentive, and weighted calculation.

The compression is calculated as shown in Equation (1).

(1) $$z_{c}=\frac{1}{H\times W}\sum_{i=1}^{H}\sum_{j=1}^{W}x_{c}\left(i,j\right)$$

Equation (1) computes the compression of the *c*-th channel of the feature, and the final result $z_{c}$ is expressed as a scalar by averaging the pool. H, W is the size of the feature map, and the current multi-scale module values of 32 × 32. $x_{c}\left(i,j\right)$ is the value of input feature function in the $\left(i,j\right)$ coordinate, representing the pixel value at the corresponding coordinate point on the feature map. In the SE attention mechanism, compression can realize global pooling, compress spatial information, effectively integrate all channel information, and lay a good foundation for the next step.

In the second step, the processed characteristic scalars need to be excited, and the calculation is shown in Equation (2).

(2) $$\;s=Sigmoid\left(W_{2}\cdot ReLU\left(W_{1}\cdot z\right)\right)$$

In the equation, $W_{1}$ and $W_{2}$ are the weights of dimension-reduced convolution and dimension-increased convolution, respectively. The dimension of feature output z is reduced and increased by these two parameters. The ReLU and Sigmoid functions are the two key activation functions in this step. After these two functions, the final channel output weight s is obtained. The Rectified Linear Unit (ReLU) function is a classical nonlinear function. It reduces the dependence between neurons and alleviates the disappearance of the gradient. The Logistic Sigmoid Function (Sigmoid) is used in this step to generate probability weights that can make the function output between (0, 1) for feature enhancement.

In the design of StarConv, the excitation step chooses to discard the fully connected structure and executes dimension reduction and dimension expansion operations on the features consecutively via grouping convolution. Through this bottleneck structure, the dependencies between channels may be learned dynamically, and reasonable picture weights can be created. Compared with the fully connected structure, the bottleneck structure has lower computing complexity. In this design, the proportion of dimension reduction is set to 0.125. After the initial convolution layer input to the multi-scale feature extraction module, the number of feature map channels is 128, then by lightweight SE feature extraction, the number of channels has realized the transformation of 128-16-128. In order to avoid that the number of input channels is so tiny that the number of compressed feature channels approaches to zero in particular specific picture classification tasks, an additional decision logic is defined in the star convolution. When the number of input channels is less than or equal to 16, no lightweight SE attention mechanism processing is performed, and only Gated Fusion and Residual Connection are kept. This method can avoid the substantial distortion of the output feature due to the loss of the feature information caused by the tiny number of compressed channels in the excitation process.

After completing the above steps, the weight of the SE attention mechanism is calculated, as shown in Equation (3).

(3) $$\hat{x_{c}}=s_{c}⊙x_{c}$$

In Equation (3), $x_{c}$ is the original feature map representation, $\hat{x_{c}}$ is the feature map representation processed by the attention mechanism, and $s_{c}$ is the weight parameter obtained by the attention mechanism. The original feature map is multiplied with the weight channel by channel, and the output feature map with attention processing is obtained after weighted processing. Through the weighted feature, the channel redundancy is alleviated and the adaptive calibration of the channel is realized.

Though the lightweight SE attention route consists of the above three phases, the result is the first output of star convolution; the other two pathways employ Gated Fusion and Residual correspondingly, and ultimately the three channel features are fused and output the result of convolution extraction.

In the Gated Fusion path, the complete input is divided into $x_{1}$ and $x_{2}$ sub-features by two sets of ordinary convolutions and then refused. Its formula is provided in Equation (4).

(4) $$\hat{x}=ReLU\left(x_{1}+g\right)⊙x_{2}$$

The ReLU function here sets the output interval to [0, 6] to prevent a gradient explosion in the computation. g is a parameter used to implement Gated Fusion computation; the initial value is set to 0 in the convolution, and the subsequent adaptive adjustment is performed depending on the change of network parameters in training. Gated Fusion is a dynamic parameter adjustment mechanism. Its function is similar to a switch. By controlling the enhancement or suppression of different features, it can realize the adaptive expression of image features, improve the propagation direction of features, and make the whole network more stable in the deep parameter update.

Residual join is an efficient module reuse approach, and the residual mapping is presented in Equation (5).

(5) $$F\left(x\right)=H\left(x\right)+x$$

In Equation (5), the original input x is added element by element to the result $H\left(x\right)$ after a series of functional transformations. In the local star convolution, the output after such processing contains both the improved features processed by many channels and the original features, which is optimized while ensuring the integrity of the global features.

After the processing of the above three channels, the distinct features are fused by a gated fusion as the final convolution result. Because the convolution output is derived from the information fusion of many channels, the number of characteristic channels is raised from 128 to 384 without further processing. At this time, the output structure of star convolution becomes too complex. As the basic component of the upcoming multi-scale feature extraction module, it will have the problem of long training time and big memory usage. Therefore, a set of 1 × 1 ordinary convolution is introduced to alter the feature channel before the formal output. This component can set different parameters according to different instances like typical convolution. In this approach, as the first portion of multi-scale feature extraction module, StarConv directly processes the input. Therefore, in this experiment, the number of input channels is fixed to 128, which not only keeps the processing results of numerous channels but also does not bring new computational burdens to the model.

In end, the structure of the full star convolution is completed. The special convolution incorporates extensive channel processing, feature fusion, and output adjustment. Compared with traditional convolution, it has more abundant parameters, can achieve more efficient feature map processing, and give more detailed and higher quality feature expression for the subsequent structure of the network.

In the multi-scale feature extraction module, the first step extracts the basic input characteristics by three distinct dimensions: 3 × 3, 5 × 5, and 7 × 7 StarConv. At this time, the output structure remains as 128 × 32 × 32 because the StarConv does not change the number of channels of the feature. After the star convolution of each dimension extracts the picture features, the feature map is down-sampled. The feature map size is lowered from 32 to 24 and the input space is reduced by 43.75%. The parameter calculation of star convolution is higher than that of standard convolution, which is beneficial to preserve memory allocation and give more processing space for dynamic routing of succeeding capsule networks.

In the multi-scale feature fusion part, the custom MFII module is employed. Multi-Feature Interaction and Integration (MFII) is a multi-scale interaction method used to aid distinct features to accomplish organic fusion after extracting multi-scale features. In the custom MFII module, there are two distinct fusion methods: cross-layer feature splicing and attention mechanism fusion. Cross-layer stitching is a horizontal feature sewing method. The main idea is to sew several features directly on the current feature dimension. Compared with the residual connection, this connection method has less requirements for the fusion structure, which is evident in the fact that it is not essential to force the same elements to be retained to achieve the addition one by one. The fused features contain not only superficial characteristics such as specific portions and textures of the image classification target but also deep information such as the contour and attitude of the full target.

In the existing MFII module, due to the huge number of channels in the multi-scale feature module, the parameters that need to be processed are complex, and the module is prone to feature splicing mistakes and partial data loss. The whole structure depends on an efficient feature fusion technique. MFII requires more features to be fused than a single star convolution. The single SE attention mechanism has the problem that the characteristics cannot be fully optimized, while the increase in computation is inevitable when shifting to a more complex attention mechanism. Therefore, in the portion of attention mechanism fusion, the ECA mechanism is added. Efficient Channel Attention (ECA) is a lightweight attention mechanism. Its core is to utilize a specific convolution to calculate the channel weight, which eliminates the whole connection computation and can achieve feature optimization with absolutely no influence on the overall calculation. The merger of these two attention mechanisms matches the needs of the upgraded capsule network.

Following feature channel processing through a sequence of convolutions, a convolutional module containing a SE attention mechanism further optimizes the features before formal output to the next module. This completes the StarConv multi-scale feature extraction module. The capsule network including this feature extraction module boosts the feature extraction capability of the model’s input stage. Multi-scale feature extraction is a method that extracts features by fusing convolutional layers of many distinct sizes.

### 2.2. Network Connectivity Module

Despite the optimization of the StarConv multi-scale module, a specific connection module must still be incorporated for structural enhancement prior to its formal integration with the capsule network.

**Figure 4:**
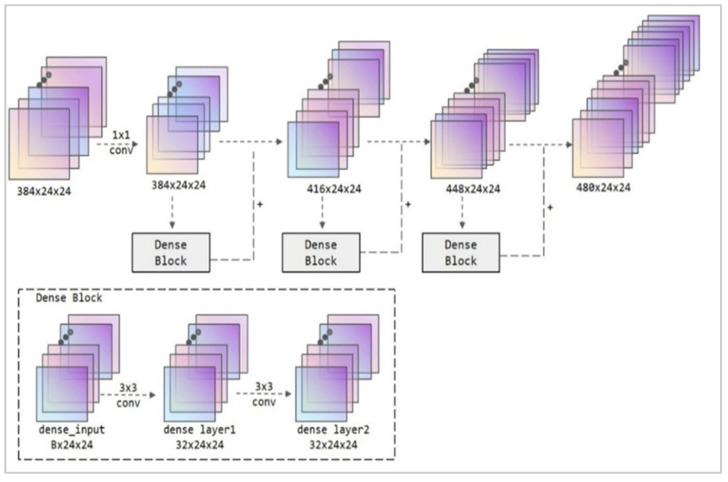
Structural diagram of the Dense Block module. The illustration depicts a 32-channel dense connection block, resulting from three dense connection procedures.

A revised network connection module, illustrated in Figure 4, was developed to enhance capsule networks. The foundational principle of this module is based on dense connections. The architecture utilizes three dense connection blocks, each comprising 32 channels, to provide the network connection layer. ReLU activation functions are employed in convolutional processing to promote feature reuse and improve the model’s overall efficiency.

Dense connectedness is a technique utilized in deep learning networks to improve feature propagation and reuse via dense skip connections. By concatenating feature maps from multiple layers, it lets low-level features immediately flow to higher layers, establishing a feedforward dense connection network structure. Each layer effectively preserves some characteristics from preceding layers, addressing the issues of disappearing gradients and inefficient feature reuse in deep networks. Due to feature reuse, the number of parameters required for learning is lowered for the same amount of feature representation, which better suppresses model overfitting and enhances generalization. This approach demonstrates more substantial effects on datasets with small pixel counts.

In this densely connected design, the Dense Block consists of two groups of 3 × 3 convolutions. Upon input entering the required nodes, the number of channels is maintained at 32. Two convolutional processes are successively employed to change the feature parameters. Compared to the original input, the output feature structure remains virtually unchanged. However, specific crucial image feature information is highlighted, facilitating further weighted image calculations and finally completing the classification function.

In the input phase of the connection module, a 1 × 1 convolution is first employed to recreate the feature map. Unlike standard pooling processes, this method better retains the spatial position information of the image, boosting the overall performance of subsequent convolutions and feature concatenation while lowering computational cost. The Dense Block further compresses the number of input channels by convolving the input channel numbers 384, 416, and 448 down to 32. After feature weighting of this input, the result is concatenated with the original input and used as input for the next stage. Finally, the network connection structure design based on Dense Block was finished. This component modifies the input of 384 × 24 × 24 to 480 × 24 × 24 (calculated as 384 + 32 × 3) by a sequence of convolutional processes. The network carries richer picture features, and the number of parameters is manageable, avoiding competition for hardware resources with multi-scale modules and capsule networks during formal training. It can serve as input for succeeding capsule networks.

This improved network connection module based on dense connection theory restores the input of the previous feature extraction module while performing more refined processing on the output. It achieves cross-layer concatenation of features, providing more feature selection references for the subsequent capsule network. Furthermore, considering the overall computational limitations of the network, the input dimensions are first constrained, and the concept of feature reuse is employed. This enhances the feature expression channels while avoiding excessive computational allocation at this stage that might impact overall performance.

### 2.3. Lightweight Optimization of Capsule Network

The lightweight capsule (Light CapsNet) implementation originates from the load reduction optimization of the dynamic routing algorithm. In capsule networks, the dynamic routing method is the translation process from main capsules to higher-level capsules, performed through several iterative parameter matrix operations. Therefore, to reduce computing costs, high-order matrices are decomposed into low-rank matrices during matrix calculations, and these low-rank matrices are subsequently employed in subsequent computations. This study approach is also applicable to the lightweight construction of capsule networks.

In matrix operations, if matrix $W\in R^{m\times n}$ has a $rank\left(W\right)=r\ll min\left(m,n\right)$, then the matrix is called a low-rank matrix. The row or column vectors in a low-rank matrix can be represented by a linear combination of a small number of basis vectors, indicating the strong correlation and redundancy in the data. In matrix operations, any matrix $W\in R^{m\times n}$ satisfies Equation (6).

(6) W=AB

In this equation, $A\in R^{m\times r}$ and $B\in R^{r\times n}$. This signifies that any matrix can be decomposed into the product of two smaller matrices. When $r=rank\left(W\right)$, this transformation is equivalent, and the original matrix’s information is perfectly preserved within the two new submatrices. When W itself is a low-rank matrix, such a decomposition can reduce the computational cost of the original matrix operation.

In deep learning, complicated models are frequently constructed from a high number of layers, in which convolution and full connection operations can be fundamentally described as large-scale matrix multiplication. Due to the redundant structure of these matrices, the matrix decomposition method can effectively reduce the parameter size and computational overhead while maintaining the performance of the model, so as to achieve efficient compression and acceleration of the model; this method or approximation method has been effectively applied in many fields and models [21,22,23]. At the same time, previous investigations have revealed that the dynamic routing process of capsule networks involves a large number of linear mapping matrices, and there is a common phenomenon of parameter redundancy between these matrices. Therefore, the inclusion of matrix factorization approach for low-rank approximation has potential optimization value, which is projected to lower the parameter size and computational complexity while retaining the performance of the model [24].

For the network layout of this experiment, there are numerous redundant parameter matrices in the digital capsule layer, and a low-rank method is employed to lower the total processing requirements of the network. Within the original parameter matrix, a transformation matrix exists. The parameters above relate to the output of the digital capsule layer and the number and dimensions of the input capsules.

In this matrix, a decomposition similar to W≈A×B has a relatively small impact on the parameter operation results themselves, but it effectively reduces the computational cost of dynamic routing. As examples using rank=4 and rank=8, the computational comparison of this improved capsule network is shown in Table 1. As shown in Table 1, matrix decomposition effectively reduces both the number of capsule parameters and the computational cost. Under the specified conditions, the number of parameters is reduced to at most half of the original, while the number of calculations is reduced to 34% of the original. By establishing proper settings to perform matrix decomposition, the design of Light CapsNet may be finished. Furthermore, when the rank parameter drops, the reduction in the number of parameters and the computational cost of the matrix will be lower.

In practical training, a smaller number of parameters is not always better because the number of model parameters is not entirely equivalent to the amount of GPU memory required. In this experiment, when rank=4, the computational cost was further optimized compared to when the value was 8. However, the training time per period rose. This is because low-rank decomposition itself requires network computation allocation, too. The computational cost optimization of capsule routing is insufficient to compensate for the higher number of parameters required by the matrix decomposition at this rank value. Therefore, given the total performance gain of the capsule network and the hardware setup, this paper eventually selects to set the rank to 8 for matrix decomposition and subsequent parameter operations.

Beyond matrix decomposition, down-sampling processes were also incorporated into the design of primary capsules to reduce computing complexity. Down-sampling lowers computing overhead by filtering out smaller parameters in the matrix, thus reducing the interference of irrelevant characteristics and lowering the dimensionality of the input space.

In the high-level capsule layer section, computational cost reduction is achieved by introducing Top-k Routing. The calculation formula for the weighted vector $s_{j}$ of the output capsule is shown in Equation (7).

(7) $$s_{j}=\sum_{i\in Top-k}c_{ij}W_{ij}u_{i}$$

**Figure 5:**
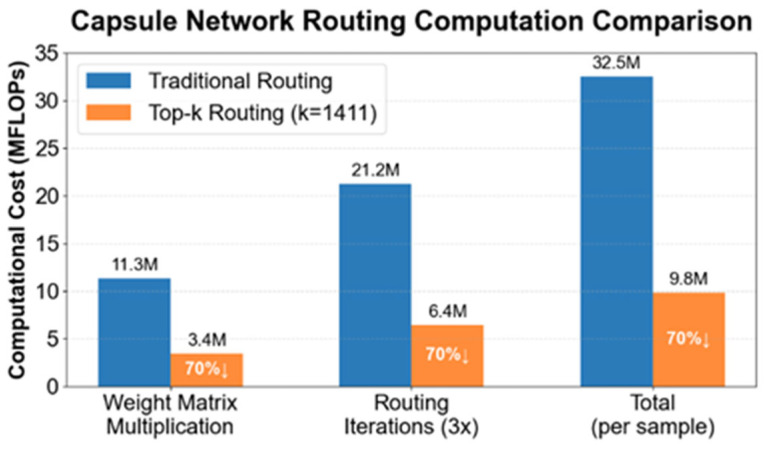
Bar chart comparing the computational cost of Top-k Routing parameters with traditional routing. When k = 1411, all parameter indicators decrease by 70%.

In this equation, $c_{ij}$ is a weighted coupling coefficient representing the probability of contribution of the current input capsule to the output capsule. $W_{ij}$ is a matrix that allows for continuous learning-based parameter transformations, enabling the mapping of input capsules to the output capsule space. $u_{i}$ represents the current input vector. The application of this method is illustrated in Figure 5.

In this article, the sparse routing parameter is set to 0.7. With 4704 primary capsules, the number of capsules actually involved in computation is reduced to 1411 (calculated as 4704 × (1 – 0.7)). Compared to traditional dynamic routing, the overall number of parameters is reduced by 70%. Multiplying each capsule by its corresponding weight coefficient enables weighted capsule feature representation. By setting a threshold k, the weight coefficients of irrelevant or low-correlation capsules can then be directly set to zero. These capsules have a minimal impact on the subsequent image feature representation. Therefore, removing these capsules reduces computational overhead, directly improving capsule layer performance. This reduction in the number of parameters lowers the memory burden of the capsule network, successfully reducing the hardware requirements for image classification, and enabling the model to use more precise training parameters to improve classification accuracy.

In the primary capsule layer, if the original parameters and convolution relations are retained, 32 entirely independent convolution layers need to be generated to express image features when the number of primary capsules is 32. In the classical capsule network, each capsule requires a separate matrix transformation during the final generation of the digital capsule from the primary capsule, i.e., in the capsule network section, the primary capsule is transformed into the digital capsule and the total number of matrix transformations in a single image classification training satisfies the number of primary capsules × the number of advanced capsules. When working with datasets like CUB_200, at least 200 digital capsules are needed because of the 200 picture types, and each transformation requires a new set of matrix transformation parameters. Therefore, although the dimension of the matrix has been lowered, the operation of the matrix transformation still has a significant overhead and is difficult to meet the experimental conditions in the face of such datasets.

In order to tackle the problem of the number of matrix transformations, the mechanism of matrix parameter sharing is introduced. By constructing a five-dimensional special matrix, the parameters of four dimensions are matched with the number of primary capsules, the dimension of primary capsules, the number of digital capsules, and the dimension type of digital capsules. The additional dimension parameter operates as a pointer that automatically sets the pointer to the relevant number of dimensions based on the presently computed dimension. In this way, the essence and procedure of matrix operation are not affected. All the transformation parameters are accumulated into a matrix, the step of dynamic computation of transformation matrix parameters is successfully omitted, and the original image classification capacity of the model is not changed.

In this study, through a variety of matrix optimization approaches, the capsule network aims to accomplish lightweight optimization. Through the foregoing optimization, the hardware needs in the model training process are lowered. Due to the vast number of images in some training datasets, the overall training time of the capsule network is still long, and the approach will be further enhanced from the part of the succeeding training strategy.

Based on the original capsule network, a temporary classification head with basic convolution and a completely linked structure is developed. The input parameters match the output of the feature extraction layer, which is 480. First, a convolution reduces the number of channels to 256, which is subsequently reduced to the matching number of classifications. For example, multi-layer convolution may be used for CIFAR-10 training to minimize feature distortion induced by extreme compression. The ultimate output channel number is ten, representing ten categories of feature expressions. This structure flattens the input feature straight to the output of the appropriate category number dimension. The structure is consistent with the enhanced lightweight capsule network. In some cases, the temporary classification head is employed as the classification result output, while in others, the fully upgraded capsule network is used to actualize the output of image classification training results.

**Figure 6:**
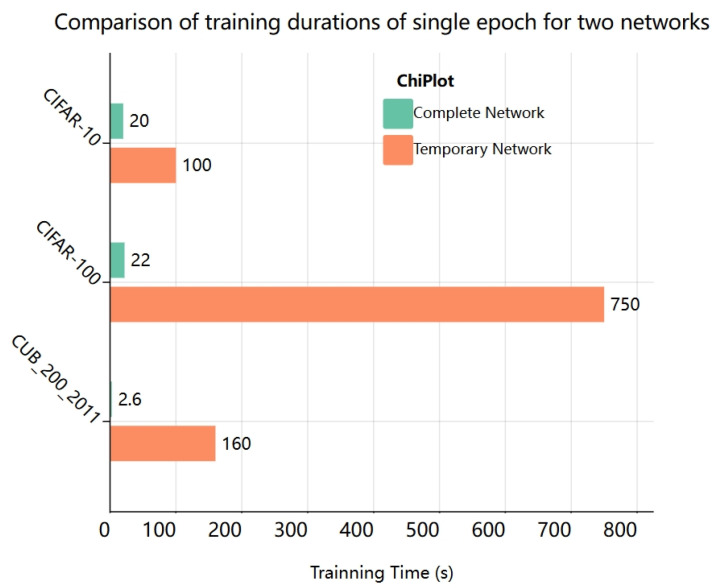
Training time for an epoch is compared under the same temporary structure and entire network circumstances.

The temporary classification header has the structure of regular convolution and a fully linked layer, which is excessively simplistic in comparison to the capsule network. In image classification, utilizing this structure as the output component may drastically save training time. Figure 6 compares the training time needed for a single epoch on different datasets. On certain datasets, the overhead is reduced by more than 90%.

In contrast to the capsule network structure, the interim classification head discards the parameter fitting in the capsule network structure to accomplish the goal of quickly fitting the feature extraction layer. Although the network composed of temporary classification heads has a quicker training speed, its feature expression ability is limited, and it is ineffective when dealing with complicated datasets. As a result, in formal image classification training, the temporary classification head might be preferred for initial training. The feature extraction layer realizes the quick fitting of parameters based on the input data at this stage of the training. In the next training, the network is replaced with a full capsule network to obtain the desired result. At this point, the model has been trained in the previous step, and the feature extraction layer parameters have been set. Compared to direct capsule network training, this training strategy can achieve a rapid improvement in early training accuracy while saving the model’s training cost in the early stage of training. The capsule network can then focus on the image classification task in the high accuracy stage.

## 3. Experiments

In order to verify the performance of the improved capsule network designed in this paper, the image classification experiments are carried out on three public datasets with different characteristics, CIFAR-10, CIFAR-100, and Cub, the basic situation of the performance of the network is observed, and the improvement of the improved network compared with the classical structure is verified. Then, in order to further verify the feasibility of the network in the practical application field, a set of image classification application experiments on the skin cancer dataset and the Forged Face dataset are introduced.

In the experiment for the first portion of the network’s fundamental performance, the image classification of the improved capsule network in this study must travel through two output paths: the feature extraction network output and the whole improved capsule network output. The experiment was split into two parts:

- Experiment with the feature Extraction Network: The first route, which includes the network and temporary structure, produces picture classification results and allows for faster parameter fitting in the feature extraction module.
- Complete Network Experiment: A second approach using the feature extraction network and Light CapsNet achieves comprehensive capsule network image classification results.

The second portion of the network application experiment uses the same network model and training strategy settings as the first, with only minor changes to the number of output categories and temporary structures.

### 3.1. Preparation

The experimental environment configuration is shown in Table 2. The experiment was divided into two phases.

Experiments to evaluate the network’s fundamental performance (Part 1) also consist of two stages. In stage 1, the Light CapsNet was frozen, and picture classification tests were carried out using just the feature extraction networks and the temporary framework. In this section of the experiment, the network settings were adjusted at 128 batches and 40 epochs. The feature extraction layer used a temporary classification head for auxiliary output, like in a traditional deep learning network design; hence, the common cross-entropy function was used. In stage 2, the temporary classification header was removed, the capsule layer was unfrozen, and ablation tests were carried out on the whole upgraded multi-scale capsule network. The batch size was smaller than in stage 1, with 64 for CIFAR-10 image classification and 32 for the other two datasets. The number of epochs was set at 150 to guarantee enough training. In stage 2, the loss function evaluated both margin loss and reconstruction loss. The margin loss is a loss function that improves performance by directly computing the distance between classification margins and giving it back to the training network for assessment. The reconstruction loss reconstructs the original picture from the high-level capsule layer’s vector output, and the difference between the reconstructed and original input images is used to adjust the model parameters. The combination of these two losses allows for more efficient capsule network training.

The improved network’s image classification experiment (Part 2) uses a more sophisticated dataset than the previous part, with more image details and content. For this portion of the experiment, leave the majority of the settings and training technique untouched. The input picture is unfolded at a higher pixel level to obtain better visual information, and to accommodate this change, the batch is stabilized at 32, and a larger epoch number, 200, is assigned to provide appropriate training. Because there is less data on both datasets, training time is lowered, and the training period of the temporary structure is set to 10.

### 3.2. Datasets

#### 3.2.1. Basic Information

The basic datasets selected for this experiment are shown in Table 3.

CIFAR-10, CIFAR-100, and CUB are usage datasets for the first part of the basic performance experiment, ranging from simple to complex and from standard icon classification to fine-grained image classification for specific application domains, and can comprehensively and meticulously evaluate and improve network performance. The ISIC derivative and EXP are part of a dataset derived from real application settings, with richer and more complex picture features. As experimental datasets, they may give more persuasive performance data for the network.

#### 3.2.2. Preprocessing

In terms of the overall dataset, ISIC and EXP have fewer photos and possess a relatively rapid training pace, while CIFAR-10, CIFAR-100, and Cub need a lengthy training period. Owing to the substantial memory overhead involved in capsule network computations, datasets employed in image experiments typically feature pixel dimensions of merely 32 pixels or even smaller. CIFAR-10 and CIFAR-100 pictures match this condition and may be sent straight into the network for training after some noise reduction processing. For datasets such as CUB, with pixel dimensions around 400 × 600, cropping is a must.

**Figure 7:**
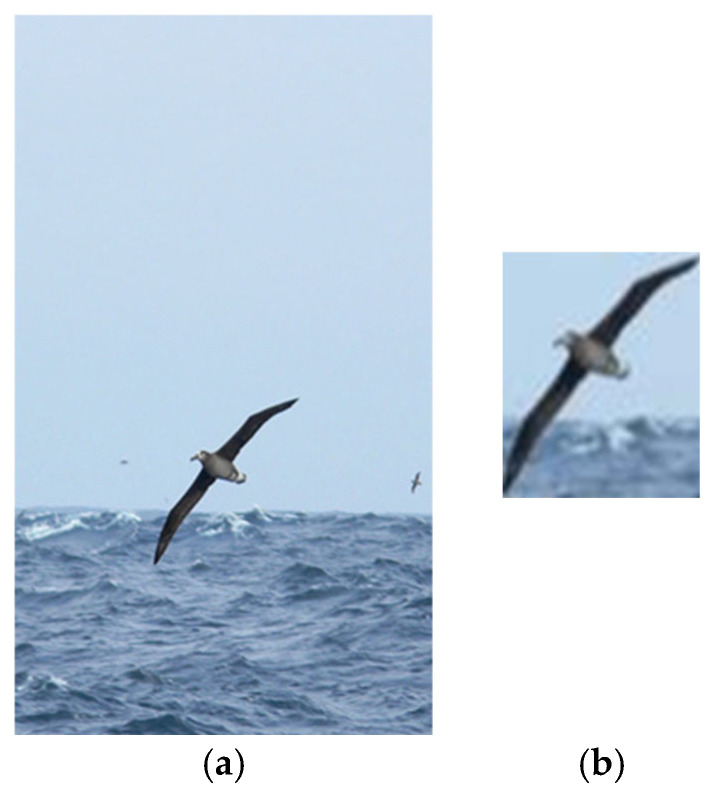
Demonstration of image cropping results for CUB-200 object recognition. (**a**) Original dataset images; (**b**) cropped image.

This article uses a pretrained object identification weight model to precisely locate the target inside an image. A 32 × 32-pixel section centered on the target is clipped and utilized as the input for a processed dataset. Because CUB is a fine-grained image classification dataset for birds, using a pretrained model capable of detecting birds is sufficient for picture cropping. When using the pretrained recognition model, the class Birds is chosen. Figure 7 shows the cropping impact on CUB photos. The picture reveals that, after the aforementioned identification and cropping, the target bird area has been mainly conserved, and the image has been successfully reduced to 32 × 32 pixels.

Following the aforementioned processing, a considerable quantity of background interference information was removed from the CUB_200 dataset photos, while also lowering image pixel count to make them suitable for capsule network image classification training. Finally, the rembg package was used to remove picture backgrounds, followed by random image alterations, which resulted in dataset augmentation to reduce overfitting in image classification.

### 3.3. Basic Performance Experiment of Improved CapsNet

Table 4 shows the fundamental parameters for this basic image recognition capability experiment. During training, the optimizer picks RAdam and sets the initial learning rate to 1 × 10^−4^, which decays gradually with the number of training epochs, using the parameter GAMMA = 0.955. The two phases use the same learning rate decay function; however, when switching between training phases, i.e., when the epoch is 41 and the temporary structure network is switched to the complete capsule network, the learning rate must be reset to 1 × 10^−4^ to ensure the effectiveness of the training across different networks. The performance metrics for the two phases will be recorded separately and utilized for further data analysis.

In phase one, the capsule layer was frozen, and only the feature extraction layer and temporary structure were utilized for image classification trials, along with the network parameters. The batch size was set to 128 and the epoch number was 40 to allow for faster fitting of the network’s feature extraction. The feature extraction layer uses a temporary classification head to aid the output result, which is part of the typical deep learning network design; therefore, the common cross-entropy function is used. In this section, 40 epochs are fixedly trained and no early stop mechanism is used.

In phase 2, the temporary sorting head was removed, the capsule layer was thawed, and ablation experiments were carried out on different components of the improved capsule network. The batch size is currently set at 64 for CIFAR-10 image classification and 32 for the classification of the other two datasets, with an epoch number of 150 to guarantee sufficient training. The capsule network’s loss function takes the form of a comprehensive evaluation of edge loss and reconstruction loss, and the design of a combination of the two can better train the capsule network while also preventing the length of the digital capsule’s activity vector from being excessively shortened during the initial stage of training. Based on the current loss of validation set, if no substantial decrease occurs within 10 epochs, training is terminated early.

To verify the experiment’s rigor and avoid unintended mistakes, in stage 2, the dataset is split five times using the same ratio to generate five distinct sets of training, validation, and test sets. Other circumstances, such as network settings and the experimental environment, are entirely consistent. Finally, the generated model’s accuracy, precision, recall, F1 value, AUC, and GM parameters are used to determine the final experimental findings. At the same time, the epoch number for the final training is maintained constant throughout the course of the experiment.

#### 3.3.1. Feature Extraction Network Experiments

In the initial stage of the experiment, the feature extraction layer experiment is carried out first to test if the feature layer has efficient feature extraction capacity, can rapidly fit certain model parameters, and can prepare for the whole capsule network experiment. In the present experiment, just the network consisting of the feature extraction module and the temporary auxiliary classification head is utilized to train the frozen capsule network.

**Figure 8:**
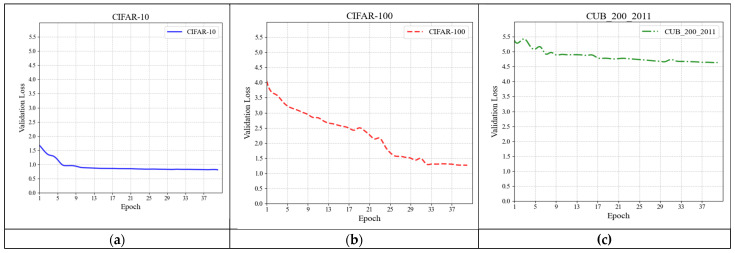
Loss curves of the Feature extraction network on validation sets of three datasets. (**a**) Loss on CIFAR-10; (**b**) loss on CIFAR-100; (**c**) loss on CUB_200_2011.

**Figure 9:**
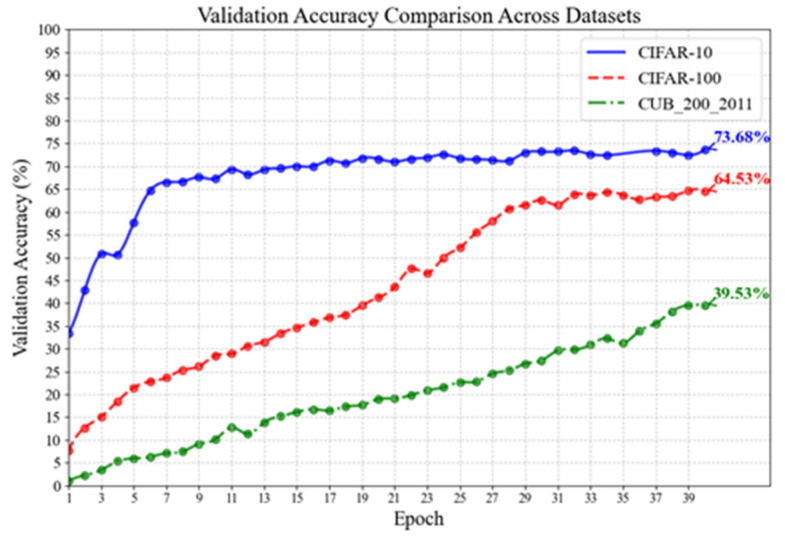
Accuracy curves of the feature extraction network on three datasets. After training for 40 epochs, the feature extraction network achieved accuracies of 73.68%, 64.53%, and 39.53% on CIFAR-10, CIFAR-100, and CUB_200_2011, respectively.

Figure 8 and Figure 9 demonstrate the experimental results of the feature extraction network on three datasets. In Figure 8, the horizontal axis indicates the number of training epochs, while the vertical axis reflects the change in loss of the feature extraction network. The loss on the validation set may be used to identify the difference between the network feature fitting parameters and the standard samples, allowing for a preliminary assessment of the network’s feature extraction and classification capabilities. The simpler dataset has fewer features and coarser detail than the complex dataset, leading in a smaller total classification loss. As shown in Figure 8, the loss for each of the three datasets follows a decreasing pattern, with a significant decline in the early stages and a progressive flattening in the later stages. Figure 8 shows the accuracy curves from this experiment. This graphic shows a direct comparison of the feature extraction network’s image classification performance on three datasets. Figure 9 indicates that picture classification accuracy for all three datasets is increasing. To facilitate speedy parameter fitting, a basic, temporary classification head was placed at the mode’s output end. Under the same training settings, the feature extraction network shows considerable differences in picture categorization accuracy. However, the mode’s image classification accuracy remains rapidly increasing across several datasets, demonstrating that this feature extraction architecture has great image classification capabilities.

In summary, across the three datasets, the feature extraction network’s validation loss decreased, with a sharp initial reduction followed by a steady flattening. The validation accuracy exhibited small changes, with an overall rising trend. This result shows that the feature extraction network can swiftly perform parameter fitting and classification tasks on a basic dataset in the early stages. However, since this experiment utilized a temporary classification head, directly mapping the retrieved picture characteristics to the output without further capsule routing filtering, inadequate network feedback led to a loss of speed after achieving a particular performance threshold. The accuracy of picture categorization by a feature extraction network is strongly associated with dataset complexity. When presented with a dataset like CIFAR-10, which contains very basic picture information and a limited number of categories, a simplified network with a frozen capsule layer nonetheless performs well in classification. However, when confronted with a dataset like CUB-200, which has many categories and few photos per category, the initial image classification accuracy is quite poor, and further training progress is slower than with other datasets.

Experiments show that this feature extraction network has a scientifically sound design and can extract features from a variety of datasets. Although the accuracy is relatively lower than that of a full image classification network owing to the simple structure of the temporary classification head in the network output, the capsule network’s parameter calculation time is much longer than that of the auxiliary classification head. Fitting this section results in fast parameter fitting of the feature extraction network, greatly decreasing the time cost of future capsule network training.

#### 3.3.2. Improved CapsNet Experiment

**Figure 10:**
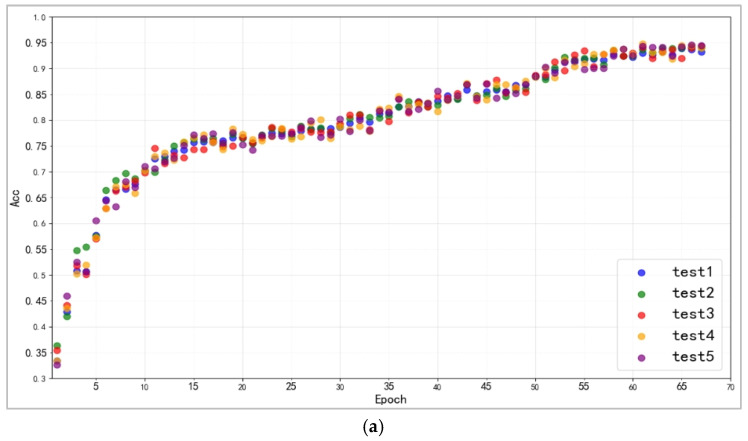

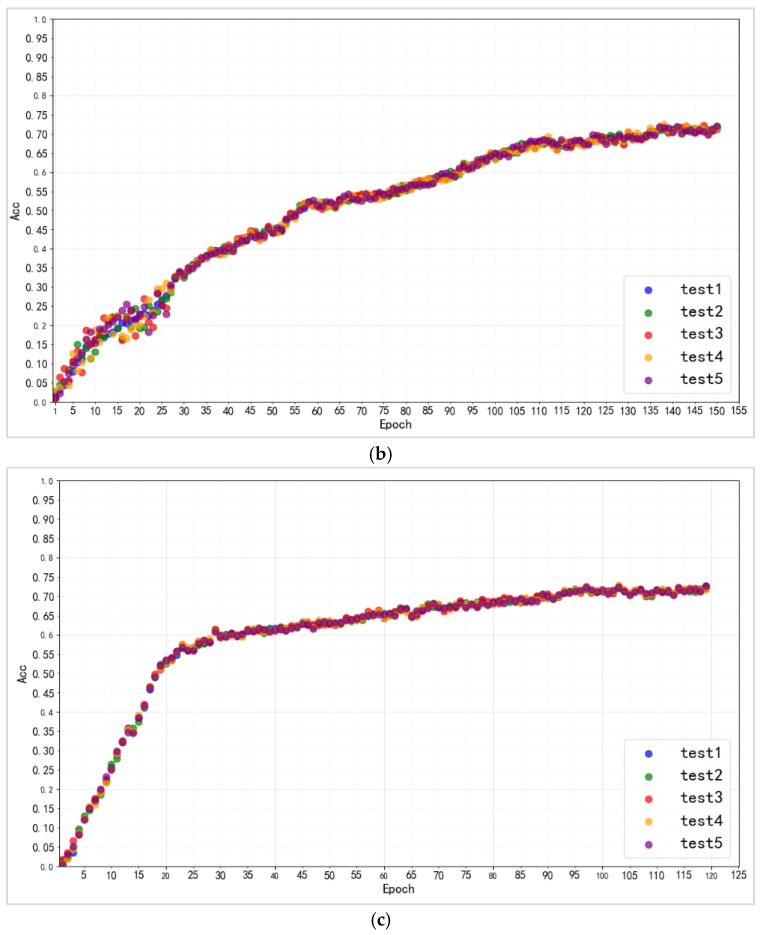
(**a**). The variation in accuracy rate of five tests on CIFAR-10. (**b**). The variation in accuracy rate of five tests on CIFAR-100. (**c**). The variation in accuracy rate of five tests on CUB_200_2011.

Building on the basic classification head tests undertaken in the first phase, we will move on to the overall enhanced capsule network studies. Each dataset is partitioned five times, and picture classification experiments are performed on each. Figure 10a–c shows scatter plots of the training outcomes of the enhanced capsule network on three datasets, using epoch as the abscissa and image classification accuracy (ACC) as the ordinate. The training result is calculated as the average value of each index from the last three datasets, as shown in Table 5. In our image classification experiments, we employed a total of six metrics to evaluate the model’s overall performance, accuracy, precision, recall, F1-score, along with AUC and GM, which can present the information provided by a confusion matrix in compact form [25]. In this way, they constitute the proper metrics to evaluate the prediction ability of a model.

Because of the pre-training of the feature extraction network, the model’s accuracy improves quickly in the early stages. The five sets of studies demonstrated a consistent rising trend overall, with no anomalous rise or drop in accuracy over the training period. According to Table 5, in the picture classification experiment, the upgraded capsule network achieves 93.81%, 72.28%, and 71.56% accuracy. The modified capsule network suggested in this study demonstrated outstanding picture classification accuracy on three datasets, and evaluation metrics such as precision (Pre), recall, and F1 evaluation values are also worth considering. On three datasets, the model’s AUC (Area Under the ROC Curve) and GM (Geometric Mean) values range from 85% to 98%, indicating that the model performs admirably from simple classification tasks to complex fine-grained image classification tasks. This demonstrates that the model has reliable image classification ability and excellent generalization.

Ablation tests were carried out on three datasets to verify the enhanced multi-scale capsule network’s performance. The experimental procedure involves gradually lowering the enhanced network design and optimization methodologies from the fully upgraded multi-scale capsule network, eventually reducing it to the traditional capsule network structure. The experiment was split into four sections.

- CapsNet: Classic capsule network;
- CapsNet + SEMS: Classic capsule network + multi-scale feature extraction module incorporating the SE attention mechanism;
- CapsNet + StarConv + SEMS: Classic capsule network + star convolution multi-scale feature extraction module;
- CapsNet + StarConv + SEMS + Dense: classic capsule network + star convolution multi-scale feature extraction module + network connectivity module based on dense connection: This section constitutes a complete multi-scale improved capsule network.

Table 6 shows the ablation experiment results for the upgraded multi-scale capsule network. The table data reveals that in the ablation experiment of the improved star convolution multi-scale capsule network, the model’s performance exhibits a steady upward trend under many techniques of optimization, and each optimization step has a beneficial influence on overall performance.

Adding several modules to the conventional capsule network design resulted in various levels of performance gain. SEMS improved the accuracy of the traditional capsule network model by 6.27%, 4.42%, and 4.13% on three datasets, respectively, with comparable numerical increases in precision, recall, and F1-score. The data above show that the multi-scale feature extraction module with the SE attention mechanism significantly improves the image classification performance of the classic capsule network, with the effect being more pronounced on datasets with fewer categories, such as CIFAR-10.

The use of StarConv resulted in further gains across all model assessment measures. Specifically, accuracy improved by 6.03%, 5.63%, and 5.6%, respectively. Precision, recall, and the total F1-score improved in comparable ways. The multi-scale feature extraction module, which included StarConv, considerably improved the model’s classification skills as compared to a normal multi-scale module. The integration of both modules improves some performance indicators. The near-equality of the four assessment measures illustrates the model’s stability and consistent performance across a variety of picture classification tasks, with accuracy, recall, and F1-score remaining within a small range. Overall, the model has greater performance.

The inclusion of the network connection block increased the accuracy of the three datasets by 5.26%, 2.43%, and 2.51%, respectively. The dense connection block is distinguished by the usage of feature reuse to reduce gradient vanishing; it is a functional module that improves, integrates, and concatenates the feature maps collected from the picture. CIFAR-10 has limited picture categories and substantial variances between them. The presently extracted features may already suit the original picture well, and the network’s need threshold for the quality of the extracted features is low, resulting in a little speed boost via feature quality optimization. However, in CIFAR-100 and CUB, the distinctions across picture categories are minimal. Even minor quality improvements after feature refinement might result in considerable performance gains.

Based on performance on a single dataset with the same network topology, the addition of SEMS, StarConv, and Dense all improved model performance. The model’s accuracy, precision, recall, and F1-score all demonstrated a consistent rising trend. However, the effect of these similar optimization modules on model structure differed amongst datasets.

Based on existing study data from three datasets [26,27], The capsule network’s accuracy on the CIFAR-10 dataset ranges between 75% and 93%. Using other models as a backbone increased the accuracy to 94.97% [28]. On the CIFAR-100 dataset, the performance of the enhanced capsule network fluctuates significantly and is maintained within the range of 43–68%, whereas the improved DeepCaps+ based on DeepCaps reaches an accuracy of 67.56% [29]. For CUB, the image size of the dataset itself cannot fit the input requirements of the capsule network well; there are relatively few related studies, and there is a lack of strong data comparison for the time being. Therefore, the focus of this paper will be on the optimization comparison with the classical capsule network. Based on the above data, the improved capsule network presented in this paper achieves higher classification accuracy across three datasets compared to other current research methods.

Using comprehensive indicators, the redesigned capsule network structure suggested in this study outperforms three datasets. In comparison to earlier research, the network on the CIFAR-10 dataset, albeit lower than the fused capsule network model, improved by more than one percentage point when compared to the enhanced structure of the single capsule network of 93%. On CIFAR-100, the model attained an accuracy of 72.28%, 4.72% better than the best of 67.56% in previous experiments. Although the CUB lacks relevant experimental data, enhanced capsule network research has resulted in a 12% gain in accuracy when compared to the conventional capsule network structure, providing additional data support for increasing capsule network performance on CUB datasets. In conclusion, when compared to earlier enhanced capsule network tests, the network proposed in this research showed significant performance increase. The final value of the model on the three datasets was much larger than the randomly assigned value of 0.5, and on a simple dataset such as CIFAR-10, the AUC of the model was higher, indicating that the model was more suitable for the large-scale dataset. On complex datasets with a large number of categories, the AUC of the model decreased slightly but remained above 87%. The findings reveal that the model maintains its existing classification accuracy and has a great overall capacity to discriminate across picture categories. For the final results of GM, this parameter is similar to the AUC under the same dataset, as well as higher on the simple dataset, and the overall remains above 85%, demonstrating that the model has good adaptability to different categories and numbers. On datasets such as CUB where there is a large imbalance of data between categories, the model can still maintain a relatively constant ability to distinguish between them.

This research introduces an enhanced capsule network that delivers performance increases across three datasets of varied categorization difficulties. The findings show that the modified capsule network proposed in this study has an advantage in picture classification, with increased accuracy and strong generalizability across databases with variable numbers of photos, image pixel sizes, and feature complexity. Using previously optimized computational data, this paper employs an improved capsule network that employs multiple methods, including multi-scale feature extraction, attention mechanisms, dense connection blocks, and matrix decomposition, to optimize memory usage and computational cost while simultaneously improving feature extraction capabilities and the model’s overall image classification performance.

### 3.4. Application Experiment of Improved CapsNet

To better assess the improved network’s viability, experiments will be repeated on two complex and practical application datasets, the skin cancer dataset and the Forged Face dataset, as well as the same set of five different training set image classification experiments under the image division. Other deep learning researchers have shared these two datasets and obtained remarkable results [30].

ISIC is an applied image dataset provided by the international collaborative group on skin imaging (ISIC) for automated detection and diagnostic studies of skin cancer, differentiating the severity of different conditions and identifying the most common types of skin cancer, which is critical for achieving scientific and efficient triage. Because various patients display the same ailment in diverse colors, forms, and sizes, and medical picture categorization is frequently difficult, it may also better evaluate the network’s and training strategy’s dependability. This study employs an extended dataset, which is a binary dataset including benign and malignant categories.

Several networks and approaches are now used in the area of picture categorization of medical skin conditions [31,32,33], but the application of capsule network in this field is still lacking. There are many kinds of skin disease datasets. For the ISIC-derived binary classification dataset used in this paper, the accuracy of the model can be maintained between 80% and 98% [34,35].

The EXP Forged Face dataset was generated by the computational intelligence and photography lab at Yonsei University’s Department of Computer Science, using photographs of human faces made by expert Photoshop designers. The photographs are so meticulously doctored, altered, and falsified that they are difficult for the naked eye to distinguish. However, the forged picture still has certain anomalous characteristics, such as an unnatural color transition and uneven pixel distribution, which may be detected by some high-performance classification methods.

In the fake face dataset, some articles have also performed corresponding research, and there are differences between different types of fake face datasets; the EXP dataset used in this paper has an overall research accuracy in the range of 60% to 94% [36,37], and the best model accuracy is 93.63%, obtained by using multi-layer CNN and stepwise training strategy [38].

According to the previous picture classification training, this experiment establishes comparable model parameters and fine-tunes several items, as shown in Table 7.

Training becomes more difficult as the picture expands, owing to the increased detail of the target dataset image, and identification issues arise for too-tiny pixels. In this experiment, the picture input to the feature extraction network was unrolled to 128 × 128, which was bigger than the previous dataset. The de-backgrounding process was the same as in the first experiment. In this experiment, the same training technique is utilized, which is to first use the temporary network and then unfreeze the whole network to finish the following training. Compared to the three datasets in the first experiment, the two datasets in this experiment, while larger in image size, are smaller in number and have shorter overall training times, showing that the two datasets in this experiment are more efficient. The improved capsule network can be trained for an epoch in 10 s, so the number of pre-training epochs is set to 10.

**Figure 11:**
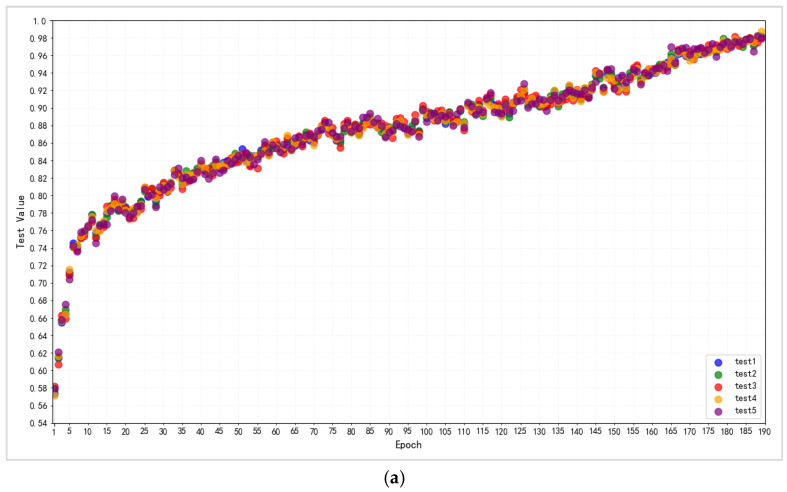

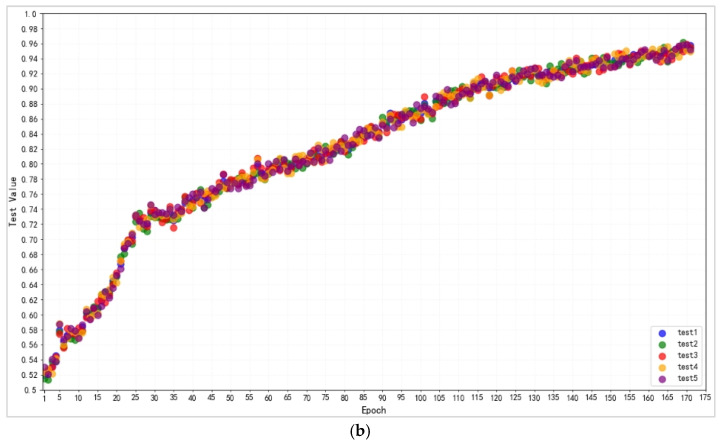
(**a**). Five groups of scatterplots of changes in experimental accuracy of improved capsule networks for ISIC-derived dermatology dataset. (**b**). Five groups of scatterplots of changes in experimental accuracy of improved capsule networks for EXP Forged Face dataset.

Finally, Table 8 and Figure 11 demonstrate the performance of this completely upgraded capsule network on two datasets. Table 8 shows the average of the final indicators. Figure 11a,b shows the accuracy scatter plot distributions for the two datasets over five different experiments.

In the ISIC dataset, the indicators indicate a steady increasing trend overall, fluctuating within a limited range; there is no evident mutation. The final upgraded capsule network scored 98.21% accuracy, 97.92% precision, 98.38% recall, and 97.96% F1-score. At the completion of the training, the AUC value was 97.98%, and the GM value was 97.78%. When compared to previous research, this result is somewhat higher than the ideal threshold, with 98.14% accuracy vs. the overall classification level of 80–98% on AUC, with a minor gap in the more advanced studies. The preceding findings demonstrate that the model performs well in the investigation of skin disease picture classification, both benign and malignant.

Although the final results of various indicators in the EXP dataset are lower than those in the ISIC dataset, they maintain a steady increase in performance, eventually achieving an accuracy of 95.38%, an accuracy of 96.07%, and a precision of 100%, 95.30% recall, and 95.54% F1-score, with final AUC and GM metrics of 96.82% and 97.51%, respectively.

The experimental findings of the above two types of application-complicated datasets demonstrate that the upgraded capsule network can play its classification advantages on certain application complex datasets, in addition to the basic test datasets, and has a higher capacity to classify. The findings also suggest that the capsule network training technique utilized in the present experiment works well with certain difficult datasets.

### 3.5. Discussion

While the upgraded capsule network shows remarkable performance increases, some testing results suggest that there is still opportunity for improvement.

In tests to improve the capsule network, the network demonstrated inadequate momentum. While model performance increased fast during the first categorization training phase, it plateaued later on. Future research will concentrate on fine-tuning the dynamic routing logic and training optimizer to better adapt other components to the picture classification objective of this upgraded multi-scale capsule network.

## 4. Conclusions

This research describes an experimental investigation on image categorization using an improved capsule network, which yielded excellent results. The particular contributions are listed below:

1. A three-channel custom StarConv structure is created to improve the performance and generalization of improved capsule networks. This convolution is utilized to create a multi-scale feature extraction module, which is successfully incorporated into the capsule network. Experiments on many datasets with various picture categories and complicated characteristics yielded greater accuracy than previous upgraded capsule networks, proving the network’s superior generalization and image classification skills.
2. A lightweight capsule network with low-rank matrices and down-sampling operations is presented to overcome high computational costs and hardware constraints. This effectively decreases capsule layer parameters by 70% and routing calculation by 33%, resulting in network computational optimization and reduced hardware needs.
3. This paper validates the feasibility of dense connectivity and matrix decomposition in capsule networks, provides supporting data, and suggests areas for future research to improve capsule networks.
4. The enhanced capsule network is trained for image classification on two datasets: ISIC derivatives and Forged Face EXP. This compensates for the absence of experimental data on certain datasets and serves as a reference for future studies.

Capsule networks continue to have significant promise for future development and study, allowing for improved optimization of computational cost and feature extraction efficiency. Improvements to capsule networks are anticipated to demonstrate more promise in future picture classification challenges and other areas of deep learning.

## Figures and Tables

**Table 1:** Comparison of parameters and flops between original matrix and low-rank decomposed matrix in dynamic routing of capsule network.

Type of matrix	Parameters (M)	FLOPs (M)	Parameters (%)	FLOPs (%)
Original matrix	120.4 M	241 M	100%	100%
Low-rank matrix (rank = 8)	120.4 M	161 M	100%	67%
Low-rank matrix (rank = 4)	60.2 M	80 M	50%	34%

**Table 2:** Experimental basic environment and network parameter settings.

Environmental Parameters	Part 1	Part 2
Operating system	Ubuntu20.04 + Python3.8	
GPU	VGPU 32 G	
Framework	PyTorch1.10.0 + CUDA11.3	
Batch size	128/64/32	32
Epoch	40/150	10/200
Loss function	Cross-Entropy Loss/ Margin Loss + Reconstruction Loss	

**Table 3:** The fundamentals of the five datasets. The features are as follows: picture dimensions in the dataset, label types, number of classes to be categorized, total number of images, dataset characteristics, and training, validation, and test set split ratios utilized in the experiment.

Datasets	Image Shape	Image Labels	Number of Classes	Number of Images	Data Characteristics	Ratio
CIFAR-10	32 × 32	Animals and Vehicles	10	60,000	Standard low-resolution images. Fundamental classification tasks.	7:2:1
CIFAR-100	32 × 32	Animals, plants, people, objects, scenery, etc.	100	60,000	More categories. Fine-grained classification tasks.	7:2:1
CUB_200_2011	About 400 × 600	Fine-grained bird species	200	11,788	Domain-specific bird images. Imbalance between categories.	5:3:2
Derivative ISIC	About 600 × 450	Skin lesions, Malignant vs. benign	2	2750	Rich in detail. Serious individual differences within the same category	7:2:1
EXP	About 600 × 600	Real or fake face	2	1200	Many ways for forgery. High requirement for network comprehensive performance	5:3:2

**Table 4:** The network training parameters used in the basic performance experiment.

Environmental Parameters	Stage 1	Stage 2
Learning rate	1 × 10^−4^	
Optimizer	RAdam	
Learning rate decay strategy	Exponential decay strategy (Resets when a phase is switched)	
Batch size	128	64 or 32
Epoch	40	150
Loss function	Cross-Entropy Loss	Margin Loss + Reconstruction Loss
Use stop strategy	N	Y

**Table 5:** The final training results of the capsule network on CIFAR-10, CIFAR-100, and Cub were improved, containing a total of six parameters, taking the average of five different dataset partitions.

Datasets	Accuracy	Precision	Recall	F1-Score	AUC	GM
CIFAR-10	93.81%	93.30%	92.85%	93.15%	96.59%	97.16%
CIFAR-100	72.28%	73.87%	72.98%	72.77%	89.81%	85.59%
CUB_200_2011	71.65%	71.47%	71.53%	71.73%	87.46%	85.64%

**Table 6:** Ablation study results of the improved multi-scale capsule network on the CIFAR-10, CIFAR-100, and CUB_200_2011 datasets.

Datasets	Model	Accuracy (%)	Precision (%)	Recall (%)	F1-Score (%)
CIFAR-10	CapsNet	76.25%	76.11%	76.24%	76.13%
	CapsNet + SEMS	82.52%	82.50%	82.52%	82.50%
	CapsNet + StarConv + SEMS	88.55%	88.78%	88.28%	88.45%
	CapsNet + StarConv + SEMS + Dense	**93.81%**	**93.30%**	**92.85%**	**93.15%**
CIFAR-100	CapsNet	59.81%	60.15%	59.58%	59.08%
	CapsNet + SEMS	64.23%	64.28%	64.14%	63.98%
	CapsNet + StarConv + SEMS	69.86%	69.94%	69.65%	69.39%
	CapsNet + StarConv + SEMS + Dense	**72.28%**	**73.87%**	**72.98%**	**72.77%**
CUB_200_2011	CapsNet	59.41%	59.89%	57.80%	59.21%
	CapsNet + SEMS	63.54%	63.41%	63.31%	63.28%
	CapsNet + StarConv + SEMS	69.14%	69.78%	69.59%	68.90%
	CapsNet + StarConv + SEMS + Dense	**71.65%**	**71.47%**	**71.53%**	**71.73%**

**Table 7:** Parameters in improved CapsNet application experiment.

Learning Rate	1 × 10^−4^
Optimizer	RAdam
Learning rate decay strategy	Exponential decay strategy
Size	128 × 128
Batch size	32
Epoch	200
Loss function	Stage 1: Cross-Entropy Loss Stage 2: Margin Loss + Reconstruction Loss
Use stop strategy	√

Note: “√” means use stop strategy. Specific instructions regarding this table are provided in the main text.

**Table 8:** Image classification results of Improved CapsNet on two datasets.

Datasets	Accuracy	Precision	Recall	F1-Score	AUC	GM
ISIC	98.21%	97.92%	98.38%	97.96%	97.98%	97.78%
EXP	95.38%	96.07%	95.30%	95.54%	96.82%	97.51%

## Data Availability

The original contributions presented in this study are included in the article. Further inquiries can be directed to the corresponding author.
